# Psychosocial Factors, Stress and Sleep Among Rural Appalachian Kentucky Residents with Type 2 Diabetes Mellitus

**DOI:** 10.13023/jah.0603.05

**Published:** 2024-10-01

**Authors:** Blake DiPaola, Zoe Taylor, Eric Hennemann, Brittany L. Smalls, Philip M. Westgate, Nancy Schoenberg

**Affiliations:** University of Kentucky; University of Kentucky; University of Kentucky; University of Kentucky

**Keywords:** Appalachia, community, diabetes, Kentucky, rural, self-management, sleep, stress

## Abstract

**Introduction:**

Rural Appalachian residents experience higher rates of most chronic diseases, including type 2 diabetes mellitus (T2DM). Stress and sleep deficiency also are common in the region.

**Purpose:**

To better understand these associated health burdens, the relationship among these conditions and psychosocial factors—such as depressive symptoms, distress, empowerment, and social support—was examined among Appalachian residents with T2DM.

**Methods:**

Using data collected from a community-based sample of Appalachian adults with T2DM, the study examined whether psychosocial factors were associated with perceived stress (Cohen Perceived Stress Scale) and self-reported sleep deficiency (Epworth Sleepiness Scale). Multilevel linear mixed effects regression modeling was used to test these associations.

**Results:**

Depressive symptoms, distress, and social support were significantly associated with perceived stress, while diabetes empowerment was not associated with perceived stress. None of these psychosocial factors were found to be associated with sleep.

**Implications:**

To our knowledge, this is the first known study to examine the relationship among psychosocial factors and perceived stress and sleep in rural Appalachian people with T2DM. With a high prevalence of mental distress in Appalachia, the findings highlight the need to further examine depression, diabetes management, and social support in people with T2DM in rural regions like Appalachia.

## INTRODUCTION

Nearly 14% of Kentucky residents have been diagnosed with Type 2 Diabetes Mellitus (T2DM), compared with 11% of the national population.^1^ Within Kentucky, T2DM is even more prevalent in the rural Appalachian Region, with 16.8% of residents being diagnosed with T2DM compared to 12.5% of those residing in non-Appalachian Regions.^2^ Multiple factors, such as stress, may increase the risk of T2DM and other conditions that elevate stress levels, including sleep disorders. Therefore, a better understanding which factors impact perceived stress and sleep patterns in Appalachian residents is needed to guide future interventions. 3,4

Research has found that numerous interrelated social determinants of health—including stress, sparse community resources, and low socioeconomic status—are associated with this elevated risk of and from T2DM. 5,6 Nearly one in five (17.9%) Kentuckians report frequent mental distress, the fourth highest statewide rate in the U.S. 5 In 2022, 82 of the 423 Appalachian counties were considered economically distressed.^6^ Low socioeconomic status remains pervasive; 12% of adults in Kentucky aged 25 and over have less than a high school education compared to 10% in the U.S. 7,8 Over 16% of Appalachian residents live below the federal poverty line compared with 12.2% nationally. 9 Such indicators of low socioeconomic status constitute stressors that are directly and indirectly associated with suboptimal T2DM management and poor health outcomes.^10^]. These factors indicate high levels of mental distress for residents of rural Appalachian Kentucky.

Two risk factors that have been implicated in T2DM are chronic stress which can lead to or exacerbate T2DM and difficulty sleeping. First, chronic stress leads to prolonged hyperglycemia through changes in glucocorticoids and catecholamines that change the metabolic system; these changes are linked to visceral fat accumulation and insulin resistance, which can ultimately cause T2DM.^11^,^12^ Stress also can complicate T2DM management, since higher stress levels are associated with lower adherence to a T2DM management plan.^13^ For example, to cope with stress, individuals may self-soothe by eating high-fat foods or drinking alcohol.^14^ Second, Appalachian residents, particularly those in Kentucky, experience a high prevalence of insomnia, which is also associated with an increase in stress levels. Although definitions vary, “insomnia” is considered the presence of an individual’s report of difficulty with sleep, generally at least three times per week. In 2021, 38.6% of Kentucky residents, one-third of whom are considered residents of Appalachia, reported experiencing sleep disorders compared with 32.3% nationally, making the state the third-highest nationally for sleep deficiency. 15,16 The Appalachian Region is a well-known insomnia “hotspot,” with 25–58% of adults indicating poor sleep quality over half of the time. Additional psychosocial factors, including depressive symptoms and social support, have been found to affect sleep quality negatively and positively, respectively [. 17,18 Extensive evidence has documented a positive association between social support and sleep outcomes.^19^,^20^ While the relationship between stress and sleep and T2DM is well established, 10,21 few studies have focused on the psychosocial factors that affect stress and sleep in the highly affected rural Appalachian Region. Therefore, this study examines the relationship among depressive symptoms, T2DM, distress, empowerment, and social support and perceived stress and sleep deficiency in Appalachian Kentucky.

## METHODS

### Study Overview

This study analyzed cross-sectional data from the “Community to Clinic Navigation to Improve Diabetes Outcomes” study (R01 DK112136, PI: Schoenberg). From the beginning, this behavioral randomized controlled intervention trial focused on community-engagement, soliciting community health priorities, selecting a relevant intervention, adapting the intervention to conform to community values and priorities, and conducting every element in partnership with community members. For example, recruitment involved partnerships with faith-based institutions, senior centers, and other community venues. Local staff people were hired and trained to serve as Community Health Workers; a Community Advisory Board met on a regular basis; and community fora were conducted every year. For this analysis, baseline data, including instruments measuring depression, diabetes distress, empowerment, social support, and perceived stress were used. The primary outcome was sleep deficiency data using the Epworth Sleepiness Scale, which consists of an eight-question tool for individuals to rank their likelihood of falling asleep while engaging in certain activities. The Office of Research Integrity at the University of Kentucky provided approval for this study (protocol #43766).

### Study Setting

This ongoing research project is taking place in six rural Appalachia counties in Kentucky. These counties were selected due to their high prevalence of T2DM and their high level of rurality determined by rural urban commuting area codes.^1^,^2^ Data were collected by local interviewers administering surveys in person inside participants’ homes, the project office, or at a mutually-agreed-upon public space. Interviews lasted between 45 and 80 minutes.

### Recruitment

As a community-engaged project, all facets of the study started and ended in the community. This orientation included the project’s focal idea, community intervention approach, participant recruitment and information dissemination. To capture the widest array of participants, including those who may not regularly obtain medical care, participants were recruited in non-clinical environments, including churches, senior centers, and other community settings throughout six rural counties. Interested individuals were eligible to participate if they were 18 years or older, a resident of Appalachian Kentucky with no plans to relocate for the next 18 months, and had a diagnosis of T2DM or a hemoglobin A1C of at least 6.5%. Additionally, interested and eligible individuals had to pass a cognitive screening to ensure the reliability of responses. To screen for cognitive impairment, the Montreal Cognitive Assessment (MoCA), a widely validated, high sensitivity tool as used. Sample items included a clock screen in which the participant reported the time, a cube copy test, and a word recall assessment. 22 To encourage participation with those with low literacy, interviewers offered to read the survey form to all participants. Indeed, to avoid stigmatizing or embarrassing, unless the participant objected otherwise, interviewers read surveys to participants.

### Measures

#### Independent Variables

*Depressive symptoms* were measured using the 20-item Center for Epidemiologic Studies Depression Scale-Revised (CESD-R). This tool is based on the DSM-IV criteria for depression diagnosis, where individuals are asked to rank how many days they experienced symptoms, ranging from ‘not at all’ to ‘nearly every day for two weeks’.^23^ A score of 16 or higher on the CESD-R indicated depressive symptoms. Therefore, depression was treated as a binary variable in statistical modeling.

*Distress* was measured using the Diabetes Distress Scale (DDS), which is a 17-question instrument. Questions are geared to assess stress associated with diabetes management (examples: not feeling confident in my day-to-day ability to manage diabetes or feeling that diabetes controls my life). Responses range from one (not a problem) to six (a very serious problem). 24 In statistical modeling, the centered, standardized value of the sum of the 17 responses was used.

*Diabetes Empowerment* was measured using the Diabetes Empowerment Scale (DES), which has eight subscales aimed at assessing the individuals’ feelings of self-efficacy relevant to their diabetes self-management. Individuals rate their feelings of empowerment on a scale from one (strongly disagree) to five (strongly agree). 25 In statistical modeling, the centered, standardized value of the sum of the eight responses was used.

*Social support* was quantified using four scales including sub-scales of companionship, informational support, ability to participate in social roles and activities as well as emotional support.^26^,^27^ This tool was sourced from the Patient-Reported Outcome Measurement Information System (PROMIS). In statistical modeling, the centered, standardized value of the sum of the sub-scales was used.

### Mediating Variable

*Perceived stress* data were obtained using the Cohen Perceived Stress Scale (PSS). This tool is a 14-question instrument where individuals score how stressful situations are in their life on a range from zero (never) to four (very often). 28 In statistical modeling, the centered, standardized value of the sum of the 14 responses was used.

### Dependent Variable

*Sleep deficiency* was assessed with the Epworth Sleepiness Scale. This eight-question tool asks individuals to rank their likelihood of falling asleep while engaging in certain activities, on a scale ranging from zero (no chance of dozing off) to three (a high chance of dozing off). 29 Researchers have used the Epworth Sleepiness Scale for more than 30 years, as it provides a subjective assessment that may reveal an undiagnosed sleep disorder including obstructive sleep apnea syndrome, narcolepsy and idiopathic hypersomnia.^30^ In statistical modeling, the centered, standardized value of the sum of the eight responses was used.

### Demographic Variables

Demographic variables included in this analysis include sex, age, marital status, education, employment status, race/ethnicity, number of people living in the home, income, subjectively assessed financial security, insurance status, smoking history, A1c, blood pressure, and health status.

### Statistical Analysis

Regression models were conducted for each independent variable to evaluate if the above-described psychosocial factors are associated with perceived stress or sleep deficiency. To test for associations, multilevel linear mixed effects regression modeling was used. To account for the potential clustering of outcomes due to the study design, these models incorporate random site effects and random household effects within sites. Perceived stress, Epworth Sleepiness Scale, distress, empowerment, and social support were centered and standardized. Each model adjusts for the demographic variables. We assessed The data were assessed via residual plots with the linear mixed model, as well as via linear regression in SAS REG in order to obtain additional approximate viewpoints of the data. For instance, variance inflation factors corresponding to the independent variables of interest were all less than two, and so multicollinearity is not a concern. The primary purpose of adjusting for demographic variables was to address the potential for confounding. Similarly, although there were outliers, they were relatively minor and uninfluential; e.g., the maximum observed Cook’s distance value was always less than 0.07.

All tests were two-sided at the 0.05 significance level, and analyses were performed in SAS version 9.4 (SAS Institute, Cary NC).

## RESULTS

Study sample characteristics (*N* = 318) are presented in Table 1**1**. Reflecting the demographics of the region, the majority of participants were white (98.4%). Most (98.1%) reported having health insurance. The average age of the sample was 63.9 years (SD = 10.5, min = 21.4, max = 88.9), Two-thirds were women and 58.4% were married. One-third of the participants reported that their highest level of education was receiving a high school diploma or GED and 30.8% indicated they sometimes struggle to make ends meet. Nearly 40% of participants were retired.^17^,^18^ The mean A1C was 7.8% (SD = 1.7).

### Depressive Symptoms

Depressive symptoms were a statistically significant and positive predictor of perceived stress (β = 0.32, SE = 0.13, *p* = .01) after adjusting for demographic variables, suggesting that depressive symptoms may moderate perceived stress **(**Table 2). In contrast, depressive symptoms were not a statistically significant predictor of sleep difficulties (β = 0.33, SE = 0.17, *p* = .06) after adjusting for demographic variables.

### Distress

Distress was a statistically significant and positive predictor of perceived stress (β = 0.18, SE = 0.05, *p* = .001) after adjusting for demographic variables. This association indicates that distress may moderate perceived stress. Conversely, distress was not a statistically significant predictor of sleep quality (β = 0.09, SE = 0.07, *p* = .20) after adjusting for demographic variables.

### Empowerment

Diabetes empowerment was not found to be a statistically significant predictor of perceived stress (β = −0.08, SE = 0.05, *p* = .09) after adjusting for demographic variables. Furthermore, diabetes empowerment was not a statistically significant predictor of sleep quality (β = −0.11, SE = 0.06, *p* = .09) after adjusting for demographic variables.

### Social Support

A lack of social support was a statistically significant predictor of perceived stress (β = −0.14, SE = 0.05, *p* = .003) after adjusting for demographic variables. Conversely, social support was not a statistically significant predictor of sleep quality (β = −0.08, SE = 0.06, *p* = .24) after adjusting for demographic variables.[Table t3-jah-6-3-44]

### Summary of Key Findings

Depressive symptoms, distress, and social support had a statistically significant and positive relationship with perceived stress in patients with T2DM. Conversely, neither depressive symptoms, distress, social support, nor empowerment had statistically significant relationships with sleep quality. Due to the lack of association between psychosocial factors and sleep quality, we did not undertake mediation assessment.

## DISCUSSION

Given the association between stress and sleep quality, the well documented prevalence of sleep difficulties, and the high chronic disease prevalence in rural Appalachian communities, it is essential to examine the potential impact of stress and other psychosocial factors within this population^6^,^16^ on diabetes onset, management, and complications. Understanding the key factors that play a role in stress and sleep of this population will inform the development of interventions to help rural residents optimize their health.

The presence of depressive symptoms was significantly associated with perceived stress. Existing research suggests a possible explanatory pathway. The neuroendocrinology of chronic stress and diagnosed depression are very similar.^29^ Furthermore, evidence suggests a link between depression and chronic stress with the prevalence and incidence of T2DM through the hypothalamic–pituitary–adrenal axis.^31^]. These study findings support further investigation to assess interventional approaches for stress and depressive symptom management in this and other high-risk populations.

Results suggest that diabetes distress is significantly related to perceived stress. Based on participants’ narratives and on existing and objective data, this finding may reflect challenges in obtaining optimal formal (health care provider) diabetes management.^32^,^33^ Study participants resided in well-documented healthcare (including primary) professional shortage areas. Indeed, the six counties in which the study was located are scored an average of 20 (on a scale of 0–26, where higher values indicate more dire shortages) for primary care on the Health Professional Shortage Area scale. Since health provider shortages are stark in Appalachian Kentucky, particularly for specialists like endocrinologists, access to regular sources of care, including routine preventive care visits, can be compromised.^34^ The lack of sufficient quantity and quality of health care access and communication may increase diabetes distress.^35^,^36^ For example, optimal communication and a positive relationship between a physician and patient are associated with lower diabetes distress and improved outcomes.^37^,^38^ Conversely, empowerment was not significantly related to perceived stress. This lack of conclusive findings suggests the need for additional studies to understand which aspects of the self-management continuum are most salient to stress.

Results suggest that overall social support is significantly related to perceived stress. Evidence suggests that a perception of social support may moderate the level of stress in a patient with T2DM.^36^ Emotional, informational, and tangible support has been shown to diminish the negative effects of stress and even depression.^40^

### Study Limitations

A modest sample size precludes us from examining all potential influences on stress, sleep, and T2DM. These psychosocial factors (depressive symptoms, distress, empowerment, social support) are not inclusive of all factors that may affect perceived stress. For example, although perceived income sufficiency was included in the analyses, economic distress was not specifically included in the analyses. Additionally, the relative homogenous population from one geographic region precludes extending the findings to other populations. For example, consistent with our region, the sample was predominantly white. Third, a possible ceiling effect may limit capacity to truly determine the effect of stress and other psychosocial factors on sleep. Fourth, methodological limitation of the Epworth Sleepiness Scale may preclude a full understanding of sleep deprivation. The Epworth Sleepiness Scale focused on sleepiness and subjective feelings of deprivation, undermining any definitive conclusions about insomnia or other sleep disorders. Finally, this study suggests multiple associations related to stress in a high-risk population, but further prospective studies are needed to determine causal relationships.

**SUMMARY BOX**

**What is already known about this topic?**

This manuscript may provide the first assessment of the relationship between multiple psychosocial factors (depression, distress, empowerment and social support) that affect perceived stress and sleep in a population of Appalachian Kentucky residents with T2DM.

**What is added by this report?**

Depressive symptoms, distress, and social support were associated with perceived stress but were not associated with sleep in patients with T2DM.

**What are the implications for future research?**

The findings provide important opportunities for future research and interventions aimed at enhancing psychosocial function, particularly among the highly affected population of rural residents with T2DM.

## Figures and Tables

**Table 1:** Baseline Sample Characteristics (N = 318)

Variable	Mean ± SD, or N (%)	N Missing
**Age (Years)**	63.9 ± 10.5	3
**Sex**		0
Female	210 (66.0%)	
Male	108 (34.0%)	
**Race/Ethnicity**		0
White	313 (98.4%)	
Black	5 (1.6%)	
**Marital Status**		3
Married	184 (58.4%)	
Divorced	51 (16.2%)	
Never Married	17 (5.4%)	
Widowed	63 (20.0%)	
**Education**		0
HS/GED	106 (33.3%)	
Associates	38 (12.0%)	
Some College	57 (17.9%)	
Bachelor	18 (5.7%)	
Graduate/Professional	99 (31.1%)	
**Employment**		0
Full-time	58 (18.2%)	
Part-time	11 (3.5%)	
Homemaker	44 (13.8%)	
Disabled	70 (22.0%)	
Retired	126 (39.6%)	
Unemployed	9 (2.8%)	
**Insurance Status**		0
Insured	312 (98.1%)	
Uninsured	6 (1.9%)	
**# of people living in home**	2.2 ± 1.1	2
**Household Income**		0
Below $10,000	46 (14.5%)	
$10,000 – $24,999	94 (29.6%)	
$25,000 – $39,999	76 (23.9%)	
$40,000 – $59,999	29 (9.1%)	
$60,000 or above	41 (12.9%)	
Not Provided	32 (10.1%)	
**Perceived Financial Status**		6
Have more than you need to live well	81 (26.0%)	
Have just about enough to get by	135 (43.3%)	
Sometimes struggle to make ends meet	96 (30.8%)	
**Self-Reported Health Conditions (“Has a dr. ever told you that you had (a)…?”)**		0
Heart attack	32 (10.1%)	
Coronary heart disease	78 (24.5%)	
Stroke	21 (6.6%)	
Kidney disease	43 (13.5%)	
High blood pressure	261 (82.1%)	
High cholesterol	225 (70.8%)	
Hepatitis	8 (2.5%)	
A1c	7.8 ± 1.7	24
Systolic Blood Pressure	141.0 ± 20.6	25
Diastolic Blood Pressure	82.8 ± 12.2	25
Number of conditions (in addition to diabetes)	2.1 ± 1.1	0
**Current Smoker?**		0
Yes	34 (10.7%)	
No	284 (89.3%)	
Perceived Stress	22.2 ± 9.4	35
Perceived Distress	28.6 ± 12.3	22
Perceived Social Support	114.7 ± 17.3	2
**Depression**		**17**
Depressed	86 (28.6%)	
Not Depressed	215 (71.4%)	
Empowerment	31.8 ± 7.1	10
Sleep Score	8.5 ± 5.6	10
Self-Report Health Status (Sum of Subscales)	447.9 ± 184.7	3
Mental health sub-scale	47.9 ± 12.7	3
Physical health sub-scale	36.5 ± 11.7	3
Diabetes Self-care Score	17.1 ± 6.2	1

**Table 2:** Adjusted Associations with Perceived Stress and the Epworth Sleepiness Scale

	Outcome: Perceived Stress		Outcome: Epworth Sleepiness Scale	
Independent Variable	Estimate (SE)	*p*-value	Estimate (SE)	*p*-value
Depressed? Yes v. No	0.32 (0.13)	0.01	0.33 (0.17)	0.06
Distress	0.18 (0.05)	0.001	0.09 (0.07)	0.20
Diabetes Empowerment	−0.08 (0.05)	0.09	−0.11 (0.06)	0.09
Social support	−0.14 (0.05)	0.003	−0.08 (0.06)	0.24

**Table 3 t3-jah-6-3-44:** Regression Model: The Association of Depression with Perceived Stress

Independent Variable	Estimate	SE	*p*-value
Depressed			
Yes	0.32	0.13	0.01
No (Reference)			

Age (years)	−0.004	0.006	0.54

Sex			
Female	−0.03	0.11	0.75
Male (Reference)			

Marital Status			0.24
Divorced	0.20	0.17	0.24
Married	0.09	0.14	0.52
Never married	0.48	0.25	0.053
Widowed (Reference)			

Education			0.18
HS/GED	0.18	0.14	0.19
Associates	0.22	0.17	0.21
Bachelor	−0.17	0.24	0.48
Graduate/professional	0.25	0.14	0.08
Some college (Reference)			

Employment Status			0.37
Disability	−0.13	0.27	0.64
Full-time	0.21	0.30	0.48
Homemaker	−0.02	0.29	0.95
Part-time	−0.25	0.35	0.48
Retired	−0.10	0.28	0.71
Unemployed (Reference)			

Financial Status			0.02
Just about enough to get by	−0.05	0.12	0.70
More than you need to live well	−0.39	0.16	0.01
Sometimes struggle to make ends meet (Reference)			

Smoking Status			
Non-smoker	−0.17	0.15	0.25
Smoker (Reference)			

Number of people living in home	−0.06	0.05	0.17

Household Income			0.64
Below $10,000	−0.14	0.22	0.53
$10,000 – $24,999	−0.10	0.19	0.59
$25,000 – $39,999	−0.21	0.19	0.28
$40,000 – $59,999	−0.31	0.22	0.15
$60,000 or above	−0.06	0.21	0.80
Not Provided (Reference)			

A1C	−0.02	0.03	0.52

Systolic Blood Pressure	−0.002	0.002	0.42

Diastolic Blood Pressure	0.0002	0.004	0.97

Insurance Status			
Uninsured	0.24	0.36	0.50
Insured (Reference)			

Race/Ethnicity			
African American	0.40	0.53	0.45
White (Reference)			

SF-36 Total Score	−0.003	0.0003	< 0.01
